# Supplementary material for: Sperm Gatekeeping: 3D Imaging Reveals a Constricted Entrance to Zebra Finch Sperm Storage Tubules
Source: Biophys J. 2019 Nov 5;117(11):2180–7. doi: 10.1016/j.bpj.2019.10.038 (PMC6895713; doi:10.1016/j.bpj.2019.10.038)
Supplement: Document S2. Article plus Supporting Material [file mmc2.pdf]

Biophysical Journal
Article

Biophysical Society

Sperm Gatekeeping: 3D Imaging Reveals a Constricted Entrance to Zebra Finch Sperm Storage Tubules

Tania Mendonca,[1,2,*] Ashley J. Cadby,[2] and Nicola Hemmings[1]
[1]Department of Animal and Plant Sciences and [2]Department of Physics and Astronomy, University of Sheffield, Western Bank, Sheffield, United Kingdom

ABSTRACT Females across many internally fertilizing taxa store sperm, often in specialized storage organs in their reproductive tracts. In birds, several hundred sperm storage tubules exist in the utero-vaginal junction of the oviduct, and there is growing evidence that sperm storage in these tubules is selective. The mechanisms underlying female sperm storage in birds remain unknown because of our limited ability to make three-dimensional, live observations inside the large, muscular avian oviduct. Here, we describe a new application of fluorescence selective plane illumination microscopy to optically section oviduct tissue from zebra finch *Taeniopygia guttata* females label free by harnessing tissue autofluorescence. Our data provide the first description of the three-dimensional structure of sperm storage organs in any vertebrate to the best of our knowledge and reveal the presence of gate-like constricted openings that may play a role in sperm selection.

SIGNIFICANCE Female birds can store sperm in microscopic tubular structures in their reproductive tract for up to several months, depending on the species. Studying these sperm storage tubules has been a major challenge because of the muscular and opaque nature of reproductive tracts in birds. We have developed a new method for imaging live reproductive tract tissue using selective plane illumination microscopy, a fluorescence microscopy technique. From these images, we could extract three-dimensional measurements of sperm storage tubules and found these structures to have a gate-like constriction, suggesting that females can actively select sperm at storage and ultimately influence the paternity of her offspring. Understanding these reproductive adaptations can help improve captive breeding programs and similar conservation strategies.

INTRODUCTION

Across many internal fertilizers, females have evolved the capacity to maintain viable sperm in specialized sperm storage organs in their reproductive tract as a strategy to maximize fertility. Sperm storage ensures the female has sufficient sperm for fertilization when copulation and ovulation are not synchronized (1). Because female promiscuity is common across taxa (e.g., birds (2), mammals (3–5), reptiles (6), fishes, (7) and insects (8–10)), storage also provides the opportunity for females to exert control over postcopulatory processes (11–13). Postcopulatory sexual selection has driven the diversification of sperm storage organs, which vary from single bean-shaped structures in damselflies (14,15) or one or more sac-like spermathecae in certain fly species (16,17) to multiple epithelial crypts in snakes (18), lizards (19), turtles (20), and birds (21,22).

In birds, epithelial sperm storage crypts are called sperm storage tubules (SSTs) and are located in the utero-vaginal junction (UVJ) of the oviduct (23). The number of SSTs possessed by a single female ranges from around 500 in the budgerigar (*Melopsittacus undulatus*) to 20,000 in the turkey (*Meleagris gallopavo*) (24). A growing body of evidence suggests that avian SSTs are an important site of sperm selection. Steele and Wishart (25) experimentally demonstrated that chicken (*Gallus domesticus*) sperm without surface membrane proteins could not enter the SSTs after normal intravaginal artificial insemination,

Submitted May 16, 2019, and accepted for publication October 22, 2019.

*Correspondence: tania.mendonca@nottingham.ac.uk or n.hemmings@sheffield.ac.uk

Tania Mendonca's present address is Department of Electrical and Electronic Engineering, University of Nottingham, University Park, Nottingham NG7 2RD, UK.

Editor: Stanislav Shvartsman.

https://doi.org/10.1016/j.bpj.2019.10.038

© 2019 Biophysical Society.

This is an open access article under the CC BY license (http://creativecommons.org/licenses/by/4.0/).

even though these sperm were capable of fertilizing the ovum when inseminated beyond the vagina and UVJ. Bobr et al. (23) also noted a lack of abnormal sperm in chicken SSTs, suggesting that abnormal sperm are unable to reach or enter sperm storage sites. The large number of SSTs present in the avian oviduct may also allow the spatiotemporal segregation of sperm from competing ejaculates (26–28).

Despite evidence that SSTs may act as a filter for high quality sperm, the mechanisms by which sperm are selected at the time of storage remain poorly understood, and how sperm enter and exit the SSTs is unknown. Froman (29) proposed a model in which sperm motility, rather than SST function, is pivotal in sperm retention in SSTs. According to this model, sperm must maintain an optimum swimming velocity to maintain their position and counter a fluid current within the SST. This model was supported by evidence that faster sperm emerged out of SSTs later than slower sperm (30) and that the passive loss of sperm from storage might be sufficient to explain last male precedence in the domestic fowl, turkeys, and zebra finches (*Taeniopygia guttata*) [(31,32); but see (27)]. However, there have been no published observations of sperm swimming inside the SSTs, and our own observations suggest sperm are not motile in storage (see Supporting Materials and Methods). Several studies have detected the presence of sperm motility suppressors such as lactic acid in Japanese quail (*Coturnix japonica*) SSTs (33), calcium and zinc in the SSTs of chicken, turkeys, and Japanese quail (34,35), and carbonic anhydrase in the SSTs of turkeys, common quail (*C. coturnix*), and ostriches (*Struthio camelus*) (36–38), and the neurotransmitter acetylcholine, released by nerve endings detected in the vicinity of SSTs (39), has been shown to enhance sperm motility (40), implying a nervous control on sperm mobilization at ejection from SSTs. Additionally, Hiyama et al. (41) presented evidence for the potential role of heat shock protein 70 (42) in enhancing sperm motility at the point of sperm release. The presence of such sperm motility suppressors and activators within or near the SSTs suggests that release of sperm from storage may not be as passive as Froman (29) suggested.

Rather than acting as passive refugia, SSTs may instead be dynamic structures, capable of active constriction and dilation to mediate the entrance and exit of sperm. Although numerous studies have failed to find smooth muscle fibers or myoepithelial cells (39,43,44) around SSTs, Freedman et al. (39) detected fibroblast-like cells and an F-actin rich cytoskeletal mesh called the "terminal web" in turkey SST epithelia. The terminal web is composed of contractile proteins (actin and myosin) and has been shown to contribute to contractility in other tissues, such as intestinal brush border cells (45,46) and embryonic pigmented epithelia in chickens (47). Freedman et al. (39) also found terminal innervations in the turkey UVJ, suggesting there may be some degree of nervous control over SST function. Recent evidence also suggests the possibility of SST contraction, influenced hormonally by the action of progesterone (48,49). It is therefore possible that the passage of sperm into and out of storage is controlled, to some degree, by the physical structure of SSTs themselves.

Our understanding of how SST structure influences sperm storage is limited by our relatively basic knowledge of SST morphology. The avian oviduct is convoluted, with opaque, muscular walls, creating numerous practical limitations for making observations of tubules in living epithelial tissue using conventional microscopy techniques. Empirical studies of SST morphology have so far used histology (23,50,51) and electron microscopy (34,43,52) on fixed tissue sections, but these approaches not only remove functional information but typically provide two-dimensional information only. Moreover, serial sectioning is laborious, and the loss of material can be difficult to avoid. Commonly used light microscopy techniques rely on thin sections and squash preparations (26,53), which are inappropriate for large tissue samples because they distort structures of interest and allow only limited imaging depths.

In this study, we developed a novel method for live, ex vivo three-dimensional (3D) imaging of SST structure using selective plane illumination microscopy (SPIM). SPIM is highly suitable for imaging large samples at cellular resolution and has lower phototoxicity levels than with other optical sectioning methods, which makes it a viable option for imaging living tissue (54,55). Using SPIM, we were able to optically section UVJ mucosal tissue up to depths of 100 μm without distorting or damaging their structure. We provide the first quantitative estimates of the 3D structure of SSTs in living tissue, including the relationship between SST length and diameter, and report the existence of a previously undescribed gate-like constriction at the entrance to tubules that may act to regulate sperm transport into and out of storage.

MATERIALS AND METHODS

Animals

This study was approved by the University of Sheffield (Sheffield, UK). All procedures performed conform to the legal requirements for animal research in the UK and were conducted under a project license (PPL 40/3481) issued by the Home Office.

Zebra finches were from a captive population kept at the University of Sheffield (56,57). Females (all between 1 and 3 years old) were placed in unisex housing for at least 2 weeks before being paired with males, in double cages (dimensions of each individual cage: 0.6 × 0.5 × 0.4 m) separated by a wire mesh with the male and female on either side. Each double cage had a modified nest box, also with a wire mesh partition, to allow both birds to enter the nest. This set up allowed the male and female to establish a normal breeding pair bond and enter breeding condition while preventing them from copulating and therefore ensuring the female had no sperm in her SSTs (sperm can be stored for up to 12 days after mating in zebra

finches (24)). Females were only included in the study once they had started to lay eggs to ensure their oviduct was in full reproductive condition. After they laid their second egg, females were euthanized (in accordance with Schedule 1 (Animals (Scientific Procedures) Act 1986)) and dissected immediately.

Sample preparation

The oviduct, including the cloaca, was immediately removed from the female, and the connective tissue surrounding it was cleared to uncoil and straighten the vagina and the UVJ. The lower end of the oviduct was cut through the middle of the uterus to obtain a segment that included the UVJ, vagina, and cloaca. This piece of the oviduct was then cut open lengthwise and pinned flat on a petri dish filled with silicone elastomer (SYLGARD 184; Dow Corning, Corning, NY). A sufficient quantity of Ham's F10 Nutrient Mix (Invitrogen, Carlsbad, CA) was added to keep the tissue moist but not submerged. For SPIM imaging, UVJ folds were cut individually with iris scissors and mounted one at a time on a custom-made sample holder (see Supporting Materials and Methods) using fine insect needles. The sample holder, with the UVJ fold mounted, was immersed in phenol-free Dulbecco's modified Eagle media/F12 media at 37°C during imaging. These conditions kept tissue viable for 1 h before it began to degrade, after which reliable data could no longer be collected.

SPIM imaging

Live UVJ tissue samples, prepared as above, were imaged using a custom-built SPIM microscope (at the University of Sheffield) with laser excitation at 473 nm and a 520-nm long-pass fluorescence emission filter (Semrock, Rochester, NY). The microscope hardware and optical components was based on the OpenSPIM platform (58) but with modifications detailed in Mendonca et al. ((59); Supporting Materials and Methods). The camera, detection, and illumination objectives and magnification were fixed for the system, ensuring that the imaging results were reproduceable. The autofluorescence image stacks were acquired using 500-ms exposure, starting at the outer surface and moving up to 100 μm deep into the tissue fold.

Characterization of autofluorescence

SSTs were clearly identifiable in live UVJ tissue during fluorescence imaging on the SPIM and had a punctate appearance on account of autofluorescent granules (Fig. 1), which appeared to be mostly confined to SST epithelial cells and were present along the entire length of the SST from orifice to blind end. No other cell structure or organelle was visible in these autofluorescence images.

To determine the organization of the autofluorescent granules in the SST epithelium, label-free images from live UVJ folds ($n = 10$ birds) were compared to those of fixed UVJ folds that had been stained for nucleic acids ($n = 3$ birds). Folds were also examined on a bright-field microscope after histological sectioning and general histochemical staining ($n = 3$ birds) (Supporting Materials and Methods).

Image analysis

The image stacks acquired using the SPIM were used to reconstruct 480 × 480 × 100 μm tissue sections containing 3D information on SST structure. Only SSTs captured entirely within the imaging volume were included. Along with a data collection time limit of 1 h per female (because of tissue degradation), this restricted our analyses to one SST per female (10 females were analyzed). SST shape information was extracted by measuring the diameter enclosed by the autofluorescence from images of live tissue at 10 equidistant points along the length of each SST.

UVJ tissue image stacks were first preprocessed in Fiji (60). Individual unbranched SSTs were selected from each female such that the entire SST structure was included in the 3D image stacks. The SSTs follow convoluted paths through the UVJ fold tissue, so to measure cross-sectional diameter at multiple points, it was necessary to slice the image volume at arbitrary angles to ensure the measurement planes were perpendicular to the direction of the SST structure. This was accomplished by first tracing

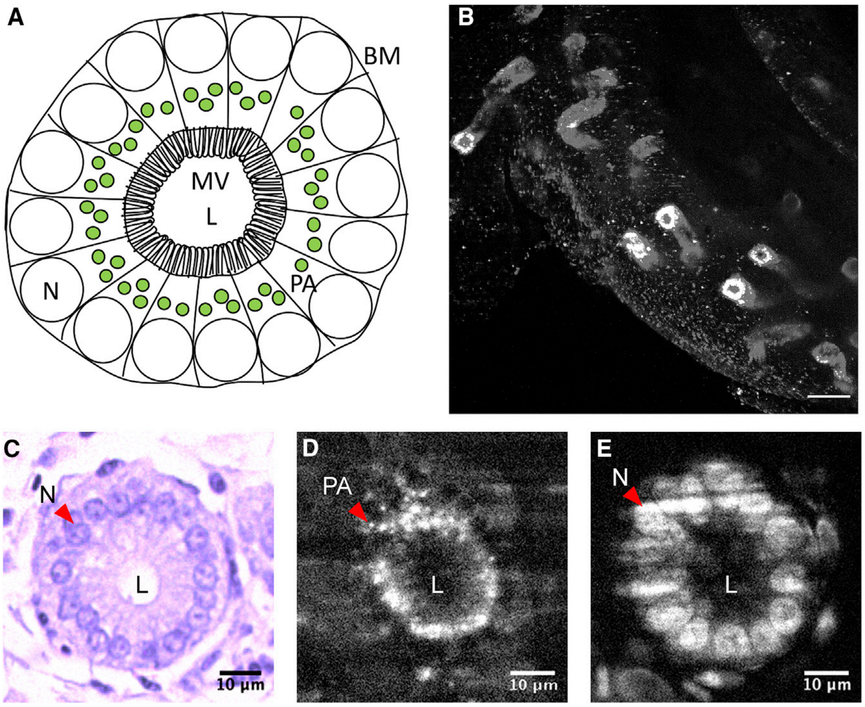

FIGURE 1 (*A*) Schematic of SST transverse section showing cellular polarization with nuclei (N) toward the basement membrane (BM) and microvilli (MV) at the apical end of the epithelium. The punctate autofluorescence (PA) detected by the SPIM is present proximal to the nucleus but not at the apical end of the epithelium near the lumen (L). (*B*) Maximal intensity projection of a UVJ fold with multiple detectable SSTs imaged on the SPIM. Scale bars, 50 μm. Measurements from (*C*) brightfield image of the cross section of SST from histology, (*D*) autofluorescence from unfixed tissue imaged on the SPIM, and (*E*) SYTO-13-labeled nuclei in fixed tissue imaged on the SPIM, respectively, were used to determine the localization of the autofluorescent granules. Scale bars, 10 μm. To see this figure in color, go online.

the direction of the SST structure using a dilated version of the SST image (generated using the "MorphoLibJ" plugin (61), followed by the application of Gaussian blur) to smooth the punctate autofluorescence. Two outlines for each SST were then semiautomatically traced in 3D from the orifice to the blind end and along opposite sides of the SST lumen using the "Simple Neurite Tracer" (62) plugin in Fiji.

The next stage of image analysis was performed using MATLAB (2015b, version 8.6; The MathWorks, Natick, MA). An average trace that passed through the SST lumen was computed from the two traces for each SST. SST lengths were measured from these average traces. For each SST ($n = 10$), the average trace was interpolated at 10 equidistant points (the first at the orifice and the 10th point before the blind end of the tubule), and at each interpolated point, a vector describing the direction of the SST at that point was computed using its nearest neighboring points on the trace (Fig. 2). By using these vectors and the interpolated points, slicing planes normal to the vectors were defined. The indices for these slicing planes were used to extract two-dimensional image sections from the undilated original image stacks using the "ExtractSlice.m" (63) function. For every extracted slice, its distance from the orifice along the luminal trace of the SST was computed using the "Arclength.m" (64) function, and the major axis diameter (d1) and the minor axis diameter (d2) of the SST (enclosed by autofluorescence) were measured.

Statistical analysis

Data analysis was performed using the statistical package R (version 3.2.3) (65). We tested whether SST diameter varied with SST length using a mixed effects model ("lmer" function from the "lme4" package (66) along with the "lmerTest" package (67)) with average SST diameter ((d1 + d2)/2) at the sampled point as the dependent variable, the distance of sampled point from SST orifice and the SST total length as fixed effects, and the bird identification as a random effect to account for repeated measures from each female.

We also assessed if the SST was elliptical or circular in cross section (the former providing greater epithelial apical surface area for increased contact with sperm) and whether any such ellipticity changed in response to SST length. A circularity index was first calculated by dividing the major axis diameter (d1) by the minor axis diameter (d2), in which a circularity index of one indicates a circular SST cross section. Data were then analyzed via a mixed effects model using the "lmer" function (66), with the circularity index as the dependent variable and the total length of the SST as a fixed effect. The sum of the major and minor axis diameters (d1 + d2) was also incorporated as a fixed effect to account for the magnitude of change in diameter along each axis as well as the distance of sampled point from SST orifice, with an interaction term between them. As before, bird identification was included as a random effect to account for repeated measures from each female.

RESULTS

The diameter of SSTs was found to be notably constricted at their orifice, suggestive of a structural "barrier" for entry and exit (Fig. 3). Beyond this constricted entrance, SSTs were largely tubular in shape, with the diameter increasing marginally along the SST's length until its midpoint, after which the diameter decreased again toward the blind end of the SST. This shape can be described by a significant quadratic relationship between SST diameter and the distance from the SST orifice (estimated effect = −16.761, $t = -3.085$, $p = 0.003$, $r^2(m) = 0.1584$, $r^2(c) = 0.4123$; Fig. 3 *A*). The relationship between SST diameter and distance from the SST orifice was also found using data from labeled tissue (Fig. S4), confirming that the shape measured from autofluorescence images was not an artifact resulting from the distribution of the autofluorescence granules. Long SSTs were neither wider nor thinner than short SSTs (estimated effect = 0.038, $t = 1.058$, $p = 0.319$).

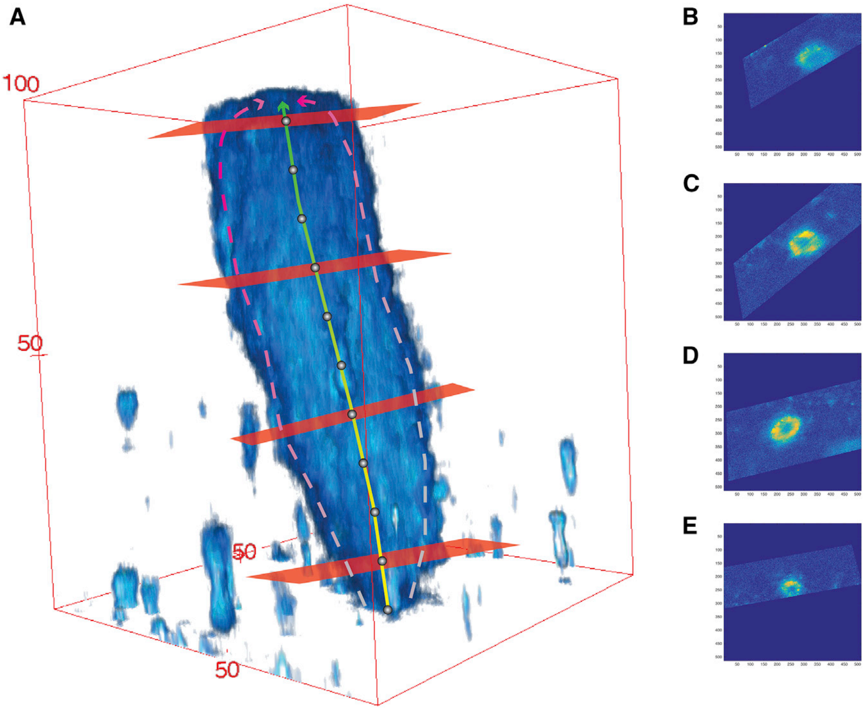

FIGURE 2 Illustration of the image analysis pipeline. (*A*) 3D rendering of an SST overlaid with traces along the sides (*dotted lines*), the computed trace through the center and slices perpendicular to the direction of the SST at four example positions (SSTs were sampled at 10 positions represented by *gray dots*). (*B–E*) Corresponding slices through the SST. Measurements were taken of the major axis and minor axis diameter for each of the slices. Axis units in pixels (converted to microns before data analysis). To see this figure in color, go online.

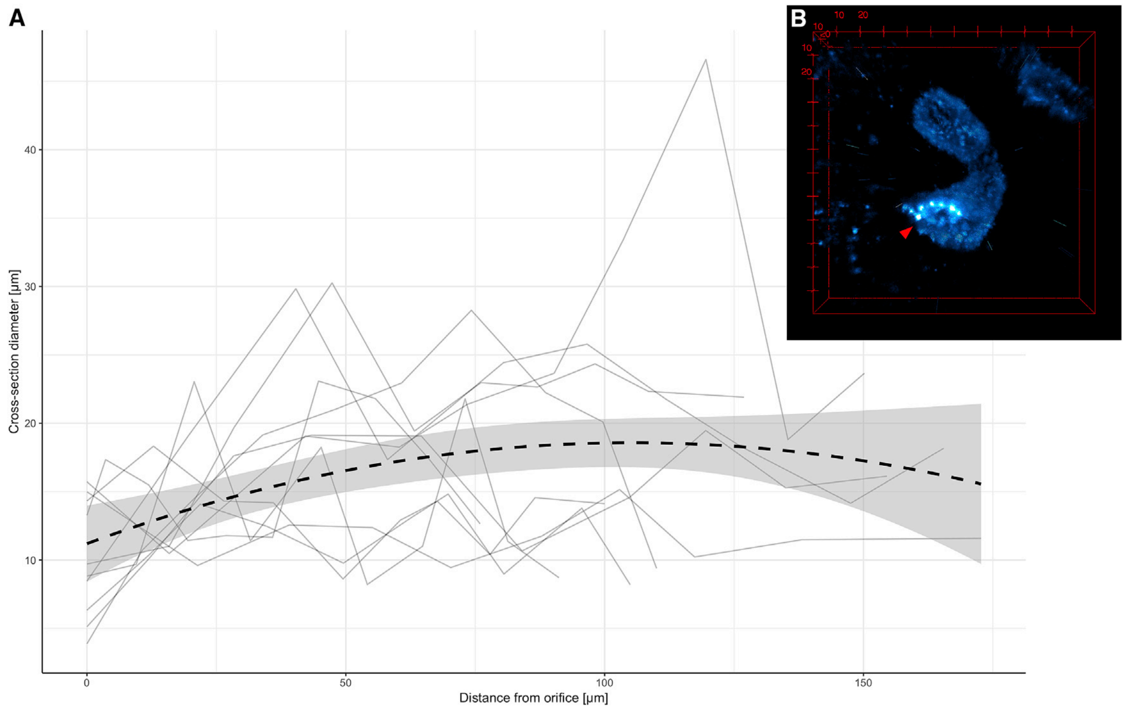

FIGURE 3 (*A*) SST diameter has a quadratic relationship with distance from the SST opening, suggesting a constriction at the orifice and a slight increase in diameter along its length up to the middle of the SST. Each plot represents measurements from an SST ($n = 10$). (*B*) 3D rendering of an SST autofluorescence signal shows its constricted orifice (*arrowhead*). Scale on red bounding box is in microns. To see this figure in color, go online.

SSTs were found to be slightly elliptical in the cross section, with the major axis diameter being 1.6 ± 0.2 times larger than the minor axis diameter. The circularity of the SST in the cross section did not vary significantly with SST diameter (estimated effect = 0.014, $t = 0.979$, $p = 0.330$), distance from orifice (estimated effect = 0.003, $t = 0.587$, $p = 0.558$), or the interaction between these two variables (estimated effect = −0.00009, $t = -0.587$, $p = 0.559$). Circularity was also not related to SST total length (estimated effect = −0.004, $t = -1.14$, $p = 0.282$).

Comparisons between the SST measurements from histology and SPIM images indicate that the autofluorescent granules are present in the supranuclear region of the SST epithelium (Fig. 1). The size of the lumen diameter scaled linearly with the width of the SST (Fig. S4), indicating that epithelial cells remained the same thickness in the cross section with increasing SST diameter. This allowed us to extrapolate shape information from the above analyses to the SST lumen, and using this method, we estimated the diameter of the SST orifice to be 3.3 ± 1.1 μm (mean ± SD; Table 1).

DISCUSSION

Using novel 3D imaging methods, we have demonstrated for the first time the existence of a constricted orifice at the entrance/exit of avian SSTs. Such a structure is likely to play an important role in sperm selection at storage. Zaferani et al. (68) recently used in vitro techniques to demonstrate how constrictions can act as gate-like selective barriers to sperm, allowing only sperm swimming above a threshold velocity to overcome the shear rate at the constriction and pass through. The narrow SST orifices we have found have a mean diameter of ~3 μm; this, with the added obstruction of microvilli (1–2 μm in length (52)), must act to restrict the rate of sperm (mean diameter at midpiece is ~0.6 μm (59)) entering and exiting the SST. We therefore propose that the constricted opening we have found in SST tubules provides a mechanism by which sperm storage and release can be regulated. This supports the idea that avian SSTs play an active and selective role in sperm storage, regulating sperm uptake and release (33–38). The constricted orifice, together with its microvilli, may act as a valve, enforcing the unidirectional

TABLE 1 SST Dimensions at Orifice and Widest Section—SST Diameter Is the Smallest at Its Orifice and Widest Near the Middle along Its Length

	Diameter (mean ± SD)	
	At Orifice (μm)	At Widest Section (μm)
Internuclear diameter[a]	12.0 ± 1.4	30.8 ± 11.1
Autofluorescence[a]	10.1 ± 4.3	16. 4 ± 6.6
Lumen diameter[b]	3.3 ± 1.1	9.1 ± 1.4

[a]Measurements acquired from SPIM image z-stacks.
[b]SST lumen diameter values were predicted from the model describing the relationship between lumen and internuclear diameter.

movement of sperm and preventing them from being flushed back out. The small luminal diameter along the SST (mean: 9 μm, Table 1) may also limit the ability of sperm to turn around inside the SST and swim out.

In terms of overall structure, we found SSTs to be slightly elliptical in the cross section, with the major axis diameter being $\sim$1.6 times larger than the minor axis diameter. This ellipticity was independent of SST radius, the distance along the SST from orifice, or total SST length. Cross-sectional ellipticity increases the surface area of the SST epithelial apical surface, allowing for a greater number of microvilli (as compared to a circular lumen with the same volume) for increased contact with sperm and an optimum exchange of nutrients and waste.

We found SST diameter to vary widely across zebra finch SSTs; nevertheless, every SST measured had its smallest diameter at its orifice. Birkhead et al. (69) suggested that some SSTs might remain inactive in the zebra finch UVJ, even in its fully developed state. It is possible that some of the variation in SST shape that we observed can be explained by the presence of functional and nonfunctional SSTs, but it is unclear whether thinner, more uniform SSTs or more distended morphs would represent the active state. Mero and Ogasawara (70) and Burke (71) described "swollen" tubules in chickens and suggested that swelling is associated with sperm release. Such swellings might help explain the outliers in our data (Fig. 3 *A*). It is possible that conformational changes in SST shape from functional to nonfunctional states may be enabled by the F-actin rich terminal web as seen in turkey SSTs (39) and caused by neural stimulation (39,72) and/or hormonal effects (48,49). Variation in SST shape might also be explained by factors not tested in this study, including age, hormone levels, and location of the SST in the UVJ.

About 4–27% of all the SSTs in the zebra finch UVJ are branched (28,73). Branched tubules were not included in our study, but individual branches are expected to show similar shapes as unbranched tubules. Hemmings and Birkhead (28) described sperm from different males differentially stored in separate branches of an SST (albeit a single observation because, in most cases, sperm from different males were stored in different SSTs). Further study of the 3D structure of branched SSTs could shed light on mechanisms that prevent sperm mixing in branched tubules.

Our novel 3D data on SST structure were made possible by the presence of punctate/granular autofluorescence, confined to the SST epithelial cells and uniformly distributed throughout the SST's entire length. These granules were found to have a supranuclear localization in the SST epithelial cells (Fig. 1). Although identifying the exact source of the autofluorescence was beyond the scope of this study, autofluorescence in a similar range has been noted in the ewe (*Ovis aries*) endometrium ($\lambda_{ex}/\lambda_{em}$ = 488/525–575 nm) (74) and in human colonic crypts ($\lambda_{ex}/\lambda_{em}$ = 488/580 nm) (75). Although such autofluorescence has been attributed to NADH metabolism in mitochondria (74,76), another likely source might be lipofuscin in lysosomes (75). Mitochondria are not confined to the apical cytoplasm of SST epithelium as observed in turkeys (77) and chickens (78), so it is unlikely that these granules represent mitochondria. Lysosomes on the other hand, are globular vesicles similar in size (<1 μm) to the autofluorescent granules observed here (79) and have been detected in the apical cytoplasm of turkey SST epithelia (77) and less abundantly in chickens (78) and passerine alpine accentor (*Prunella collaris*) (79). Multiple studies have also detected the presence of acid phosphatase, an enzyme found in lysosomes in the supranuclear cytoplasm of SST epithelia in turkeys (21), quail (80), chickens (81), and ducks (*Anas sp.*) (82), but not in the SST lumen, which corresponds with the autofluorescence pattern we observed here in the zebra finch. Acid phosphatase has been implicated in autolysis associated with oviduct regression (83) as well as with sperm release (82). If this is true, the label-free imaging methods developed here may provide exciting new means for investigating SST functional development throughout the reproductive cycle. Identifying the chemical nature of the autofluorescent substance present in SST granules therefore represents an important avenue for future research.

In summary, we have demonstrated that sperm storage structures in living vertebrate oviductal tissue can be imaged label-free using SPIM microscopy, and this novel 3D imaging technique has enabled us to produce the most detailed account of avian SST structure to date, including the discovery of a previously undescribed gate-like constriction at the entrance/exit of tubules that is likely to act as a key selective barrier. The imaging methods described here hold immense potential for studying in vivo sperm storage and sperm-female interactions.

Data supporting this study is available through figshare under accession numbers 10.6084/m9.figshare.10295204 (data tables and R data analysis code) and 10.6084/m9.figshare.10295447 (MATLAB scripts for SST image analysis).

SUPPORTING MATERIAL

Supporting Material can be found online at https://doi.org/10.1016/j.bpj.2019.10.038.

AUTHOR CONTRIBUTIONS

T.M. coordinated the study, conducted the experiments and analyses, and wrote the manuscript. N.H. conceived the study, conducted the sperm motility experiment, and advised on data analyses. All authors participated in the design of the study, helped draft the manuscript, and gave final approval for publication.

ACKNOWLEDGMENTS

The authors are grateful to Prof. Tim Birkhead and Prof. Simon Jones from the University of Sheffield for discussion and advice early on in the study. Thanks to Mark Kinch from Skelet.AL and Phil Young, Lynsey Gregory,

Emily Glendenning, and Jamie Thompson from the University of Sheffield for technical assistance.

The work was supported by Imagine: Imaging Life project at the University of Sheffield, which was funded by the MRC (MR/K015753/1). N.H. was supported by a Royal Society Dorothy Hodgkin Fellowship (DHF160200).

REFERENCES

1. Birkhead, T. R. 1992. Sperm storage and the fertile period in the Bengalese Finch. *Auk.* 109:620–625.
2. Birkhead, T. R., and A. P. Møller. 1995. Extra-pair copulation and extra-pair paternity in birds. *Anim. Behav.* 49:843–848.
3. Hanken, J., and P. W. Sherman. 1981. Multiple paternity in Belding's ground squirrel litters. *Science.* 212:351–353.
4. Tegelström, H., J. Searle, …, S. Mercer. 1991. Multiple paternity in wild common shrews (*Sorex araneus*) is confirmed by DNA-fingerprinting. *Heredity.* 66:373–379.
5. Amos, W., S. Twiss, …, S. S. Anderson. 1993. Male mating success and paternity in the grey seal, Halichoerus grypus: a study using DNA fingerprinting. *Proc. Biol. Sci.* 252:199–207.
6. Uller, T., and M. Olsson. 2008. Multiple paternity in reptiles: patterns and processes. *Mol. Ecol.* 17:2566–2580.
7. Avise, J. C., A. G. Jones, …, J. A. DeWoody. 2002. Genetic mating systems and reproductive natural histories of fishes: lessons for ecology and evolution. *Annu. Rev. Genet.* 36:19–45.
8. Gromko, M. H., D. G. Gilbert, and R. C. Richmond. 1984. Sperm transfer and use in the multiple mating system of Drosophila. *In* Sperm Competition and the Evolution of Animal Mating Systems. R. I. Smith, ed. Academic Press, pp. 371–426.
9. Moritz, R. F. A., P. Kryger, …, S. Tingek. 1995. High degree of polyandry in Apis dorsata queens detected by DNA microsatellite variability. *Behav. Ecol. Sociobiol.* 37:357–363.
10. Crozier, R. H., and E. J. Fjerdingstad. 2001. Polyandry in social Hymenoptera - disunity in diversity? *Ann. Zool. Fenn.* 38:267–285.
11. Birkhead, T. R., and A. P. Møller. 1998. Sperm Competition and Sexual Selection. Elsevier Science, Amsterdam, the Netherlands.
12. Simmons, L. W. 2001. Sperm Competition and its Evolutionary Consequences in the Insects. Princeton University Press, Princeton, NJ.
13. Eberhard, W. G. 1996. Female Control: Sexual Selection by Cryptic Female Choice. Princeton University Press, Princeton, NJ.
14. Córdoba-Aguilar, A., E. Uhía, and A. C. Rivera. 2003. Sperm competition in Odonata (Insecta): the evolution of female sperm storage and rivals' sperm displacement. *J. Zool. (Lond.).* 261:381–398.
15. Siva-Jothy, M. T. 1987. The structure and function of the female sperm-storage organs in libellulid dragonflins. *J. Insect Physiol.* 33:559–567.
16. Ward, P. I. 1993. Females influence sperm storage and use in the yellow dung fly Scathophaga stercoraria (L.). *Behav. Ecol. Sociobiol.* 32:313–319.
17. Pitnick, S., T. Marrow, and G. S. Spicer. 1999. Evolution of multiple kinds of female sperm-storage organs in Drosophila. *Evolution.* 53:1804–1822.
18. Fox, W. 1956. Seminal receptacles of snakes. *Anat. Rec.* 124:519–539.
19. Conner, J., and D. Crews. 1980. Sperm transfer and storage in the lizard, *Anolis carolinensis*. *J. Morphol.* 163:331–348.
20. Gist, D. H., and J. M. Jones. 1989. Sperm storage within the oviduct of turtles. *J. Morphol.* 199:379–384.
21. Zavaleta, D., and F. Ogasawara. 1987. A review of the mechanism of the release of spermatozoa from storage tubules in the fowl and Turkey oviduct. *World's Poult. Sci. J.* 43:132–139.
22. Bakst, M. R. 1987. Anatomical basis of sperm-storage in the avian oviduct. *Scanning Microsc.* 1:1257–1266.
23. Bobr, L. W., F. W. Lorenz, and F. X. Ogasawara. 1964. Distribution of spermatozoa in the oviduct and fertility in domestic birds. I. Residence sites of spermatozoa in fowl oviducts. *J. Reprod. Fertil.* 8:39–47.
24. Birkhead, T. R., and A. P. Møller. 1992. Numbers and size of sperm storage tubules and the duration of sperm storage in birds: a comparative study. *Biol. J. Linn. Soc.* 45:363–372.
25. Steele, M. G., and G. J. Wishart. 1996. Demonstration that the removal of sialic acid from the surface of chicken spermatozoa impedes their transvaginal migration. *Theriogenology.* 46:1037–1044.
26. King, L. M., J. P. Brillard, …, A. M. Donoghue. 2002. Segregation of spermatozoa within sperm storage tubules of fowl and Turkey hens. *Reproduction.* 123:79–86.
27. Briskie, J. V. 1996. Spatiotemporal patterns of sperm storage and last-male sperm precedence in birds. *Funct. Ecol.* 10:375–383.
28. Hemmings, N., and T. Birkhead. 2017. Differential sperm storage by female zebra finches *Taeniopygia guttata*. *Proc. Biol. Sci.* 284:20171032.
29. Froman, D. 2003. Deduction of a model for sperm storage in the oviduct of the domestic fowl (*Gallus domesticus*). *Biol. Reprod.* 69:248–253.
30. Froman, D. P., T. Pizzari, …, T. R. Birkhead. 2002. Sperm mobility: mechanisms of fertilizing efficiency, genetic variation and phenotypic relationship with male status in the domestic fowl, Gallus gallus domesticus. *Proc. Biol. Sci.* 269:607–612.
31. Birkhead, T. R., J. Pellatt, and F. M. Hunter. 1988. Extra-pair copulation and sperm competition in the zebra finch. *Nature.* 334:60–62.
32. Birkhead, T. R., and J. D. Biggins. 1998. Sperm competition mechanisms in birds: models and data. *Behav. Ecol.* 9:253–260.
33. Matsuzaki, M., S. Mizushima, …, T. Sasanami. 2015. Lactic acid is a sperm motility inactivation factor in the sperm storage tubules. *Sci. Rep.* 5:17643.
34. Holm, L., H. Ekwall, …, Y. Ridderstråle. 2000. Localization of calcium and zinc in the sperm storage tubules of chicken, quail and Turkey using X-ray microanalysis. *J. Reprod. Fertil.* 118:331–336.
35. Bakst, M. R., and M. P. Richards. 1985. Concentrations of selected cations in Turkey serum and oviductal mucosae. *Poult. Sci.* 64:555–563.
36. Holm, L., and Y. Ridderstråle. 1998. Localization of carbonic anhydrase in the sperm-storing regions of the Turkey and quail oviduct. *Histochem. J.* 30:481–488.
37. Holm, L., and G. J. Wishart. 1998. The effect of pH on the motility of spermatozoa from chicken, Turkey and quail. *Anim. Reprod. Sci.* 54:45–54.
38. Holm, L., S. D. Ruziwa, …, Y. Ridderstråle. 2000. Carbonic anhydrase in the utero-vaginal junction of immature and mature ostriches. *Br. Poult. Sci.* 41:244–249.
39. Freedman, S. L., V. G. Akuffo, and M. R. Bakst. 2001. Evidence for the innervation of sperm storage tubules in the oviduct of the Turkey (*Meleagris gallopavo*). *Reproduction.* 121:809–814.
40. Atherton, R. W., C. M. Cisson, …, T. K. Golder. 1980. Quantitation of avian spermatozoan motility: neurochemical regulation. *Gamete Res.* 3:17–24.
41. Hiyama, G., M. Matsuzaki, …, T. Sasanami. 2013. Sperm activation by heat shock protein 70 supports the migration of sperm released from sperm storage tubules in Japanese quail (*Coturnix japonica*). *Reproduction.* 147:167–178.
42. Lindquist, S., and E. A. Craig. 1988. The heat-shock proteins. *Annu. Rev. Genet.* 22:631–677.
43. Van Krey, H. P., F. X. Ogasawara, and J. Pangborn. 1967. Light and electron microscopic studies of possible sperm gland emptying mechanisms. *Poult. Sci.* 46:69–78.
44. Gilbert, A. B., M. E. Reynolds, and F. W. Lorenz. 1968. Distribution of spermatozoa in the oviduct and fertility in domestic birds. V. Histochemistry of the uterovaginal sperm-host glands of the domestic hen. *J. Reprod. Fertil.* 16:433–444.

45. Hirokawa, N., T. C. Keller, III, …, M. S. Mooseker. 1983. Mechanism of brush border contractility studied by the quick-freeze, deep-etch method. *J. Cell Biol.* 96:1325–1336.

46. Keller, T. C., III, K. A. Conzelman, …, M. S. Mooseker. 1985. Role of myosin in terminal web contraction in isolated intestinal epithelial brush borders. *J. Cell Biol.* 100:1647–1655.

47. Owaribe, K., R. Kodama, and G. Eguchi. 1981. Demonstration of contractility of circumferential actin bundles and its morphogenetic significance in pigmented epithelium in vitro and in vivo. *J. Cell Biol.* 90:507–514.

48. Ito, T., N. Yoshizaki, …, T. Sasanami. 2011. Progesterone is a sperm-releasing factor from the sperm-storage tubules in birds. *Endocrinology.* 152:3952–3962.

49. Hemmings, N., T. R. Birkhead, …, S. Briere. 2015. Timing associated with oviductal sperm storage and release after artificial insemination in domestic hens. *Theriogenology.* 83:1174–1178.e1.

50. Bakst, M. R. 1998. Structure of the avian oviduct with emphasis on sperm storage in poultry. *J. Exp. Zool.* 282:618–626.

51. Van Drimmelen, G. C. 1946. "Spermnests" in the oviduct of the domestic hen. *J. S. Afr. Vet. Med. Assoc.* 17:42–52.

52. Bakst, M. R., and G. Bauchan. 2015. Apical blebs on sperm storage tubule epithelial cell microvilli: their release and interaction with resident sperm in the Turkey hen oviduct. *Theriogenology.* 83:1438–1444.

53. Bakst, M. R. 1992. Observations on the Turkey oviductal sperm-storage tubule using differential interference contrast microscopy. *J. Reprod. Fertil.* 95:877–883.

54. Huisken, J., J. Swoger, …, E. H. Stelzer. 2004. Optical sectioning deep inside live embryos by selective plane illumination microscopy. *Science.* 305:1007–1009.

55. Reynaud, E. G., U. Krzic, …, E. H. Stelzer. 2008. Light sheet-based fluorescence microscopy: more dimensions, more photons, and less photodamage. *HFSP J.* 2:266–275.

56. Birkhead, T. R., E. J. Pellatt, …, H. Castillo-Juarez. 2005. Genetic effects on sperm design in the zebra finch. *Nature.* 434:383–387.

57. Bennison, C., N. Hemmings, …, T. Birkhead. 2015. Long sperm fertilize more eggs in a bird. *Proc. Biol. Sci.* 282:20141897.

58. Pitrone, P. G., J. Schindelin, …, P. Tomancak. 2013. OpenSPIM: an open-access light-sheet microscopy platform. *Nat. Methods.* 10:598–599.

59. Mendonca, T., T. R. Birkhead, …, N. Hemmings. 2018. A trade-off between thickness and length in the zebra finch sperm mid-piece. *Proc. Biol. Sci.* 285:20180865.

60. Schindelin, J., I. Arganda-Carreras, …, A. Cardona. 2012. Fiji: an open-source platform for biological-image analysis. *Nat. Methods.* 9:676–682.

61. Legland, D., I. Arganda-Carreras, and P. Andrey. 2016. MorphoLibJ: integrated library and plugins for mathematical morphology with ImageJ. *Bioinformatics.* 32:3532–3534.

62. Longair, M. H., D. A. Baker, and J. D. Armstrong. 2011. Simple neurite tracer: open source software for reconstruction, visualization and analysis of neuronal processes. *Bioinformatics.* 27:2453–2454.

63. Teng, P. 2011. Extract Slice from Volume. MATLAB Central File Exchange, July 27, 2019 https://uk.mathworks.com/matlabcentral/fileexchange/32032-extract-slice-from-volume.

64. D'Errico, J. 2012. arclength. MATLAB Central File Exchange, July 27, 2019 https://uk.mathworks.com/matlabcentral/fileexchange/34871-arclength.

65. R Development Core Team. 2015. R: A Language and Environment for Statistical Computing. R Foundation for Statistical Computing, Vienna, Austria https://www.r-project.org/.

66. Bates, D. M., M. Mächler, …, S. C. Walker. 2015. Fitting linear mixed-effects models using lme4. *J. Stat. Softw.* 67:1–48.

67. Kuznetsova, A., P. B. Brockhoff, and R. H. B. Christensen. 2017. lmerTest package: tests in linear mixed effects models. *J. Stat. Softw.* 82:1–26.

68. Zaferani, M., G. D. Palermo, and A. Abbaspourrad. 2019. Strictures of a microchannel impose fierce competition to select for highly motile sperm. *Sci. Adv.* 5:eaav2111.

69. Birkhead, T. R., J. E. Pellatt, and F. M. Hunter. 1990. Numbers and distribution of sperm in the uterovaginal sperm storage tubules of the zebra finch. *Condor.* 92:508–516.

70. Mero, K. N., and F. X. Ogasawara. 1970. Dimensions of uterovaginal sperm-storage tubules of the chicken and their possible significance in sperm release. *Poult. Sci.* 49:1304–1308.

71. Burke, W. H. 1968. Release of spermatozoa from storage sites in the hen's oviduct. PhD thesis. University of California, Davis.

72. Gilbert, A. B., and P. E. Lake. 1963. Terminal innervation of the uterus and vagina of the domestic hen. *J. Reprod. Fertil.* 5:41–48.

73. Birkhead, T. R., and F. M. Hunter. 1990. Numbers of sperm-storage tubules in the Zebra Finch (Poephila guttata) and Bengalese Finch (*Lonchura striata*). *Auk.* 107:193–197.

74. Druart, X., J. Cognié, …, J. L. Gatti. 2009. In vivo imaging of in situ motility of fresh and liquid stored ram spermatozoa in the ewe genital tract. *Reproduction.* 138:45–53.

75. DaCosta, R. S., H. Andersson, …, B. C. Wilson. 2005. Autofluorescence characterisation of isolated whole crypts and primary cultured human epithelial cells from normal, hyperplastic, and adenomatous colonic mucosa. *J. Clin. Pathol.* 58:766–774.

76. Brookner, C. K., M. Follen, …, R. Richards-Kortum. 2000. Autofluorescence patterns in short-term cultures of normal cervical tissue. *Photochem. Photobiol.* 71:730–736.

77. Schuppin, G. T., H. P. Van Krey, …, G. B. Meyer. 1984. Ultrastructural analyses of uterovaginal sperm storage glands in fertile and infertile Turkey breeder hens. *Poult. Sci.* 63:1872–1882.

78. Burke, W. H., F. X. Ogasawara, and C. L. Fuqua. 1972. A study of the ultrastructure of the uterovaginal sperm-storage glands of the hen, *Gallus domesticus*, in relation to a mechanism for the release of spermatozoa. *J. Reprod. Fertil.* 29:29–36.

79. Chiba, A., and M. Nakamura. 2001. Microscopic structure of the sperm storage tubules in the polygynandrous alpine accentor, Prunella collaris (Aves). *Acta Zool.* 82:299–306.

80. Renden, J. A., E. B. May, and F. H. Benoff. 1981. Histochemistry of uterovaginal sperm-host glands in Japanese Quail (*Coturnix coturnix* japonica) with reference to the period of oviposition. *Poult. Sci.* 60:2529–2535.

81. Gilbert, A. B., M. E. Reynolds, and F. W. Lorenz. 1968. Distribution of spermatozoa in the oviduct and fertility in domestic birds. 8. The effect of a foreign object in the uterus on secretions of the sperm-host glands and the survival of spermatozoa in the oviduct of the domestic hen. *J. Reprod. Fertil.* 17:311–314.

82. Pal, D. 1977. Histochemistry of the utero-vaginal junction with special reference to the sperm-host glands in the oviduct of the domestic duck. *Folia Histochem. Cytochem. (Krakow).* 15:235–242.

83. Heryanto, B., Y. Yoshimura, …, T. Okamoto. 1997. Involvement of apoptosis and lysosomal hydrolase activity in the oviducal regression during induced molting in chickens: a cytochemical study for end labeling of fragmented DNA and acid phosphatase. *Poult. Sci.* 76:67–72.

Biophysical Journal, Volume *117*

Supplemental Information

Sperm Gatekeeping: 3D Imaging Reveals a Constricted Entrance to Zebra Finch Sperm Storage Tubules

Tania Mendonca, Ashley J. Cadby, and Nicola Hemmings

Part 1: Tissue preparation and comparison of live and fixed tissue

Sample Mounting

Conventional sample mounting methods with SPIM involve embedding samples in agarose or alternatively, using adhesives or hooks to grip large tissue samples. Embedding the tissue would block access to SST orifices making it inappropriate for studying sperm-female interactions. Moreover, gripping UVJ tissue with hooks or adhesives was found to be unsuitable in preliminary tests due to the flexible nature of the tissue, which resulted in unwanted tissue movement during imaging scans. To overcome this problem, a modified sample holder was designed for suspending utero-vaginal (UVJ) tissue in the sample chamber as follows:

The bottoms of free-standing micro-centrifuge tubes were cut to a depth of 5 mm. 1 mL syringes were cut to remove the dispensing tip and the micro-centrifuge tube pieces were fixed at right angles to the syringes using epoxy glue (Figure S1). The well of the micro-centrifuge tube was filled with silicone elastomer (SYLGARD® 184; Dow Corning). UVJ tissue samples and/or individual UVJ folds could be mounted on to the surface of the silicone elastomer using the ends of fine insect needles. The free end of the syringe was fastened on the sample arm of the SPIM positioning system for imaging.

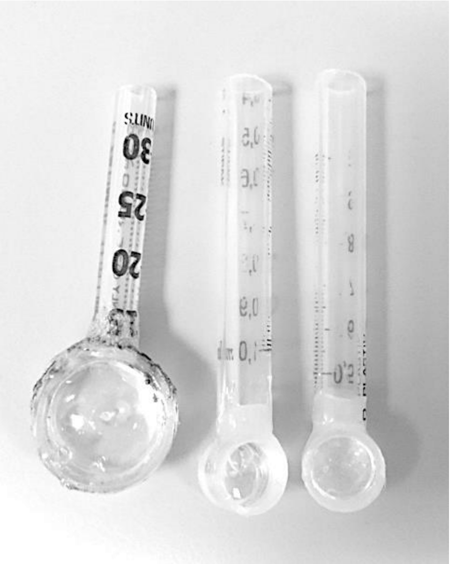

Figure *S1*: Customised sample holders of two sizes for imaging oviduct tissue samples. The tissue samples were pinned on the silicone elastomer surface with the UVJ mucosal surface and the SST orifices exposed.

Localisation of autofluorescence granules

Live UVJ folds (n = 10 birds) imaged using the SPIM were compared to those of fixed UVJ tissue folds imaged, on the SPIM after staining for nucleic acids (n = 3 birds), and on a bright-field microscope after general histochemical staining (n = 3 birds).

For histochemical examination, pieces of fixed UVJ tissue from three females were sent to the Skeletal Analysis Laboratory (skelet.AL) at the University of Sheffield for processing. The tissue was set in resin blocks and 3 µm thick sections were cut using a microtome. The tissue sections were stained with haematoxylin to label nucleic acids and eosin to label the cytoplasm and other acidophilic structures. The sections were mounted on individual slides and micrographs were taken of the slides on a microscope with bright-field (Leica DMBL with Infinity 3 camera, Luminera Corporation) at 250X magnification using a tiling method. Multiple overlapping

image tiles with a 50% overlap were taken for each slide. The individual image tiles were then stitched together using the 'TrakEM2' plugin [1] in Fiji [2] to digitally reconstruct each histological section.

10 random SST transverse sections from the histology slides for each bird (n = 3 birds) were measured using Fiji [2]. Two measurements were recorded for each (1) the lumen diameter; (2) the diameter between SST nuclei (referred to as 'inter-nuclear diameter' from here on), and (3) the diameter bound by the basement membrane (the 'outer diameter') of the SST from each cross-section (Figure S2). The diameter of the SST from autofluorescence images and the inter-nuclear diameter from SPIM images were measured as outlined in the main manuscript text.

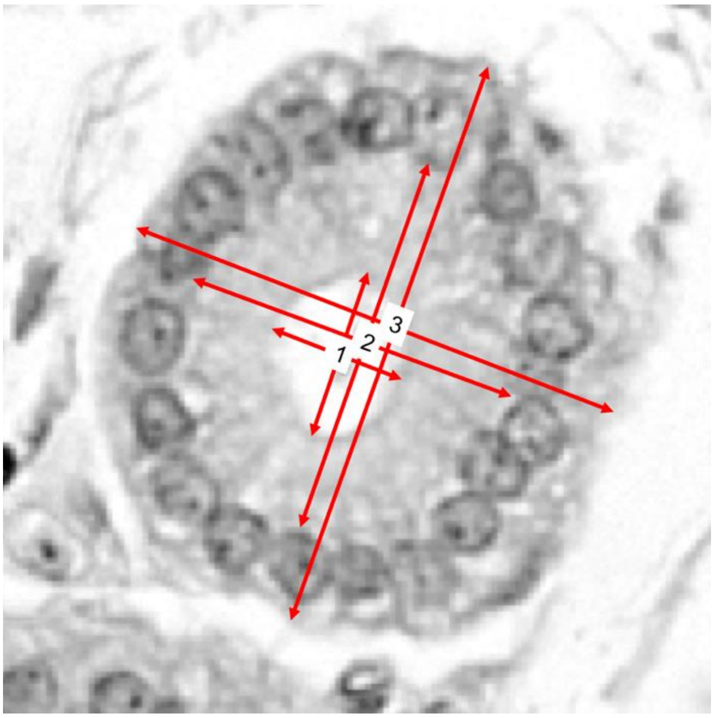

Figure S2: Cross section of an SST from histology with arrows indicating the diameters measured from the images: **1.** lumen diameter, **2.** the inter-nuclear diameter and **3.** outer diameter of the SST.

The three different diameter measurements from the histology images showed a linear correlation (lumen and inter-nuclear diameter (log transformed): estimated effect = 1.1339, t = 5.479, p = <0.0001) suggesting that lumen size scales proportionately with the size of the SST epithelial cells (Figure S3). Changes in the diameter of autofluorescence could therefore be considered to be representative of changes in SST lumen size, so the three-dimensional structure of live SSTs could be analysed from the autofluorescence images.

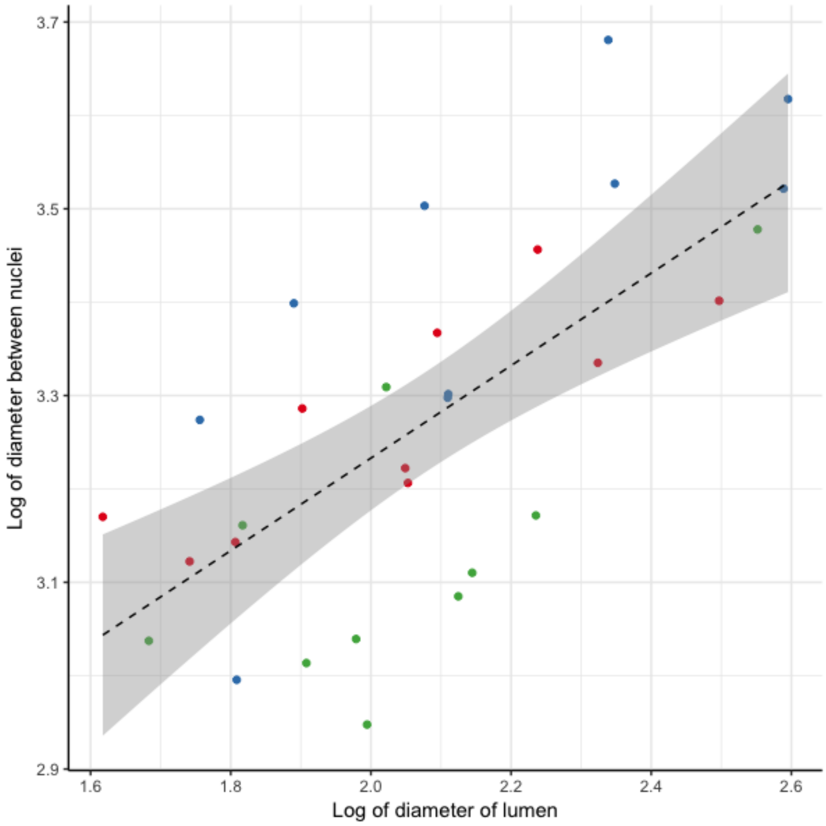

Figure S3: Diameter of SST lumen is positively correlated with diameter between SST epithelial nuclei and the outer diameter of the SST. Measurements were recorded from histology images (n = 3 birds, with 10 transverse sections measured per bird). Different colours have been used for data points from each individual.

Table 1: Diameter measurements [μm] from SST sections

	n (Birds*)	Diameter (mean ± S.D.)
**lumen from histology**	3	8.3 ± 2.3 μm
**autofluorescence from SPIM**	10	16.1 ± 6.6 μm
**inter-nuclear diameter from SPIM**	3	22.9 ± 7.7 μm
**inter-nuclear diameter from histology**	3	26.9 ± 5.3 μm
**outer diameter of SST from histology**	3	40.2 ± 5.7 μm

*Data are based on 10 randomly selected SST transverse sections per individual.

Validation with labelled fixed tissue

After 2-3 folds had been removed for live tissue imaging, the remaining UVJ tissue was flooded with 5% formalin while still pinned flat on the silicone elastomer and left to fix for two hours at room temperature. The fixed tissue samples were then transferred to micro-centrifuge tubes with 1 mL of 5% formalin for storage at room temperature. For imaging fluorescently stained SST epithelia, individual folds were cut from the fixed UVJ tissue of three females and incubated with 100 μL solution of 10 μM SYTO 13 nucleic acid stain (Molecular Probes Inc., UK) in PBS overnight at room temperature in the dark. The labelled folds were then mounted on the sample holder, one at a time, in the same way as the live tissue samples, and imaged in phenol free DMEM/F12 on the SPIM using the same settings as with live tissue (above), but with 1 ms exposure time.

Image analysis was performed and major axis (d1) and minor axis (d2) diameter measurements were recorded as described for the live tissue image stacks.

A quadratic mixed effects model with average SST diameter [(d1+ d2) / 2] at the sampled point as the dependent variable, the distance of sampled point from SST orifice and the SST total length as fixed effects, and the bird ID as a random effect showed a similar relationship as with measurements from live tissue, between inter-nuclear diameter and distance from SST orifice (estimated effect = -25.293, t = -4.530, p = 0.0001, Figure 12) confirming that the autofluorescence has a uniform distribution and is a fair proxy for analysing SST shape in label-free images (Figure S4).

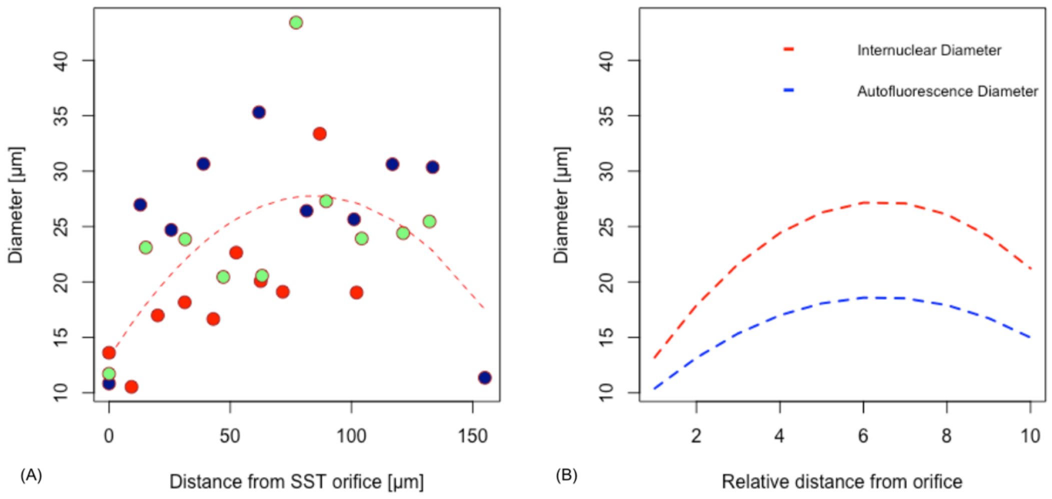

Figure S4: The inter-nuclear diameter shows the same relationship with distance from orifice as the autofluorescence diameter. **(A)** Each colour represents data from a SST. **(B)** Fits to experimental data from label-free images of SSTs (blue, n = 10) and labelled fixed SSTs (red, n = 3).

Model selection for SST diameter to length relationship

The data show a quadratic trend as seen from the plots in Figure 3 and Figure S4. A linear relationship between SST diameter and distance from orifice was also tested and was found to be statistically significant [estimated effect: 15.29221, t = 2.712, p = 0.00804, r^2(m) = 0.1009, r^2(c) = 0.3538]. This was not as strong as the quadratic relationship reported [estimated effect = -16.761, t = -3.085, p = 0.003, r^2(m) = 0.1584, r^2(c) = 0.4123]. The above models were compared by performing a Chi-square test using the 'anova' function in R which showed a better fit for the quadratic relationship (p = 0.0023; smaller Akaike's Information Criteria (AIC) and Bayesian information criteria (BIC) values for the quadratic regression model: linear model- AIC = 646.64, BIC = 659.66; quadratic model- AIC = 639.36, BIC = 654.99). Higher order polynomial regressions were also tested in R, the results from which show that the quadratic effect was still the most statistically significant. The results have been reproduced below:

Linear mixed model fit by REML. t-tests use Satterthwaite's method ['lmerModLmerTest']

Formula: Diameter ~ Polynomial (Distance from orifice, 5) + Total length of SST+ (1 | Bird ID)

	Estimate	Std. Error	df	t value	p
(Intercept)	10.98587	4.96486	8.57267	2.213	0.05565 .
1st order (linear)	15.24785	5.67588	85.14802	2.686	0.00868 **
2nd order (quadratic)	-16.71283	5.46547	85.67117	-3.058	0.00297 **
3rd order (cubic)	4.39748	5.33776	85.85596	0.824	0.41231
4th order	-5.05563	5.34529	85.96753	-0.946	0.34690

(quartic)					
5th order (quintic)	2.93449	5.33739	85.98189	0.550	0.58388
Total length of SST	0.03813	0.03607	8.61638	1.057	0.31924

Part 2: Sperm motility inside, and after release from, the sperm storage tubules of zebra finches

As part of a separate study (by NH), 13 female zebra finches that had copulated with males were dissected to isolate sperm storage tubules (SSTs) for sperm counts using standard light microscopy. During this work, we took the opportunity to observe whether sperm appeared to be motile, both inside and outside the SST.

The females were dissected as described in the main text of the paper, but instead of mounting utero-vaginal folds for SPIM imaging, folds were dissected on a microscope slide under a Nikon SMZ25 stereomicroscope to isolate and open individual SSTs and release sperm following methods described in Hemmings & Birkhead (2017). A single SST containing a large number of sperm (mean = 33 per SST) was isolated per fold (46 SSTs in total), submerged on a microscope slide in warmed nutrient media (Ham's F10: Invitrogen, UK; typically used for avian sperm motility analysis), which was then observed on a heated stage (38º C) using 200X phase contrast microscopy. Each SST was observed for 5 mins to screen for sperm motility inside the tubule. After this initial observation period, the SST was broken open using fine dissection needles and sperm were released. The slide was then observed for another 5 mins on the heated stage to screen for sperm motility after release from the tubule.

Across at total of 1520 sperm from the SSTs of 13 different females, we did not observe any motile sperm either inside, or after release from, the SSTs. This suggests that sperm are not motile during storage and require some form of activation to

trigger motility on release. However, we cannot rule out the possibility that the techniques used for isolating SSTs and extracting sperm may have affected sperm motility, and since the study was not originally designed to test this idea, we do not have data on or observations of stored sperm using other techniques and/or at other places in the oviduct. Our observations should therefore be treated as preliminary and interpreted with care.
